# Association between osteoarthritis and the incidence of Parkinson’s disease in the United Kingdom

**DOI:** 10.1016/j.prdoa.2021.100120

**Published:** 2021-11-24

**Authors:** Louis Jacob, Lee Smith, Ai Koyanagi, Alexis Schnitzler, Jae Il Shin, Karel Kostev

**Affiliations:** a Research and Development Unit, Parc Sanitari Sant Joan de Déu, Dr Antoni Pujadas, 42, Sant Boi de Llobregat Barcelona, Spain; b Centro de Investigación Biomédica en Red de Salud Mental (CIBERSAM), Madrid, Spain; c Faculty of Medicine, University of Versailles Saint-Quentin-en-Yvelines, Montigny-le-Bretonneux, France; d Centre for Health, Performance and Wellbeing, Anglia Ruskin University, Cambridge, UK; e Institució Catalana de Recerca i Estudis Avançats (ICREA), Pg. Lluis Companys 23, Barcelona, Spain; f Université de Paris, Inserm U1153, Epidemiology of Ageing and Neurodegenerative Diseases, Paris, France; g Department of Pediatrics, Yonsei University College of Medicine, Seoul, Republic of Korea; h Epidemiology, IQVIA, Frankfurt, Germany

**Keywords:** Osteoarthritis, Parkinson’s disease, Retrospective cohort study, United Kingdom

## Abstract

**Background:**

Little is known on the potential relationship between osteoarthritis and Parkinson’s disease.

**Objective:**

Therefore, the objective of this retrospective cohort study was to analyze the association between osteoarthritis and the incidence of Parkinson’s disease in patients followed up for up to 10 years in general practices in the United Kingdom.

**Methods:**

This study included patients diagnosed for the first time with osteoarthritis in one of 256 general practices in the United Kingdom between 2000 and 2016 (index date). Patients without osteoarthritis were matched (1:1) to those with osteoarthritis using propensity scores based on sex, age and index year. In individuals without osteoarthritis, index date corresponded to a randomly selected visit date. The outcome of this study was the 10-year cumulative incidence of Parkinson’s disease in patients with and without osteoarthritis. Cox regression analyses were adjusted for common comorbidities.

**Results:**

This study included 260,224 patients (62.0% women; mean [SD] age 66.4 [12.7] years). The 10-year cumulative incidence of Parkinson’s disease was 1.2% in patients with osteoarthritis and 0.6% in their counterparts without osteoarthritis (log-rank p-value < 0.001). The adjusted Cox regression model further showed a positive and significant association between osteoarthritis and the incidence of Parkinson’s disease (HR = 1.82, 95% CI: 1.63–2.02). Similar results were obtained in all sex and age subgroups.

**Conclusions:**

In this retrospective cohort study conducted in the United Kingdom, there was a positive association between osteoarthritis and the incidence of Parkinson’s disease. More research is warranted to confirm or refute these findings in other settings and countries.

## 1 Introduction

Parkinson’s disease is a progressive neurodegenerative disorder caused by a loss of dopaminergic neurons [1], and characterized by motor (e.g., resting tremor, muscular rigidity and bradykinesia) and non-motor symptoms (e.g., apathy, orthostatic hypotension and olfactory dysfunction) [2], [3]. The global prevalence of Parkinson’s disease is 0.1–0.2% [4], and the number of patients affected by Parkinson’s disease has steadily increased over the past decades [5]. Frailty [6] and mortality rates [7] are higher in people with Parkinson’s disease than in the general population, while treatment and management of this neurological condition impose substantial costs on patients and their relatives [8], [9]. Thus, future research should aim to identify risk factors for and correlates of Parkinson’s disease to inform targeted interventions.

One potential risk factor for Parkinson’s disease that has been understudied is osteoarthritis [10], [11]. Osteoarthritis is a common degenerative joint disease frequently affecting the hand, hip and knee [12], and involving several pathological changes such as progressive destruction of articular cartilage, osteophyte formation and subchondral bone thickening [13]. A retrospective cohort study of 66,720 individuals from Taiwan showed a positive association between osteoarthritis and the risk of Parkinson’s disease (adjusted hazard ratio [HR] = 1.41, 95% confidence interval [CI]: 1.16–1.70), and the relationship between the two chronic conditions was particularly strong in the subgroup of participants with hip or knee osteoarthritis [11]. The association between osteoarthritis and Parkinson’s disease may involve common risk factors such as inflammation [14], [15] and vitamin D deficiency [16], [17], [18], as well as several mediators such as physical inactivity [19], [20], hypertension [21], [22] and depression [23], [24]. Although the previous study conducted in Taiwan has advanced the field [11], its findings may not be generalizable to other countries. Indeed, Taiwan is one of the countries with the lowest age-standardized incidence and prevalence of osteoarthritis, while it is one of the only two countries for which the rate of years lived with disability for osteoarthritis has decreased over the past decades [25]. In this context, more research focusing on the osteoarthritis-Parkinson’s disease relationship in other regions of the world is warranted.

Therefore, the goal of this retrospective cohort study, including 260,224 patients followed up in 256 general practices from the United Kingdom for up to 10 years, was to investigate the potential association between osteoarthritis and the incidence of Parkinson’s disease.

## 2 Methods

### 2.1 Database

Data from the Disease Analyzer database (IQVIA) were used for this study. The Disease Analyzer database contains demographic, clinical and pharmaceutical data obtained in anonymous format on a regular basis from primary care practices in the United Kingdom [26]. Clinical data are coded using the International Classification of Diseases, 10th revision (ICD-10), while pharmaceutical data are coded using the Anatomical Classification of Pharmaceutical Products of the European Pharmaceutical Market Research Association (EphMRA). Several criteria (e.g., completeness of documentation and linkage between clinical and pharmaceutical data) are used to assess the quality of the data imported in the database. Finally, practices included in the Disease Analyzer database are selected based on multiple variables (i.e., age of physician, community size category and region) to obtain a representative sample of all practices in the United Kingdom [26].

### 2.2 Study population

**Fig. 1:**
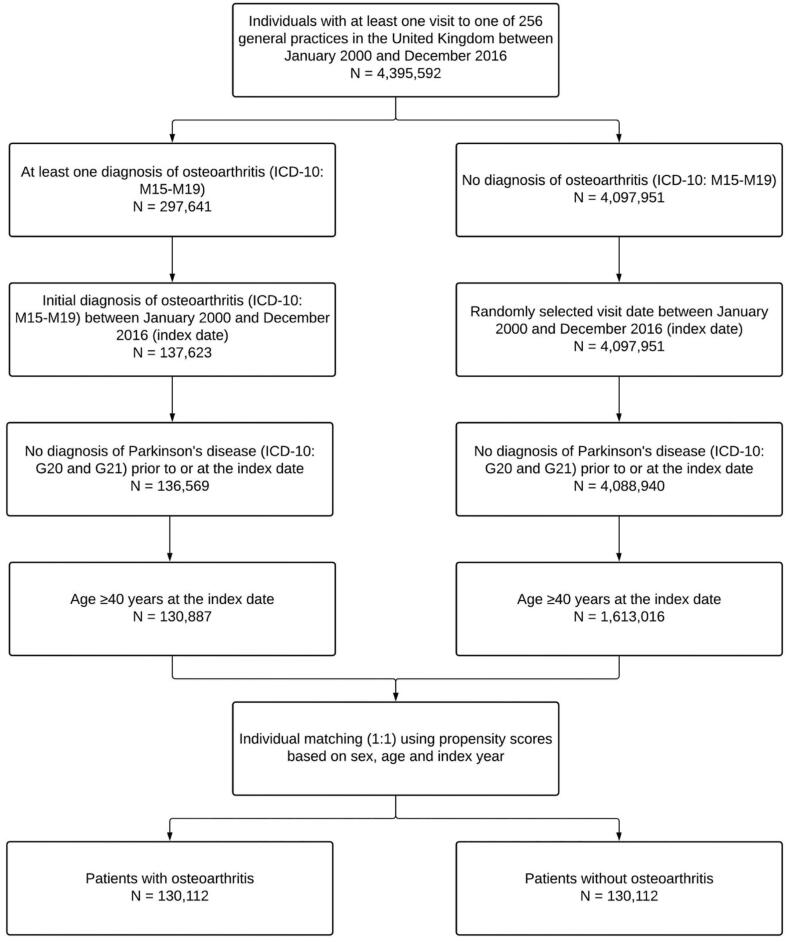
Selection of study patients.

This retrospective cohort study included patients diagnosed for the first time with osteoarthritis (ICD-10: M15-M19) in one of 256 general practices in the United Kingdom between January 2000 and December 2016 (index date). To be included, patients had to have received no diagnosis of Parkinson’s disease (ICD-10: G20 and G21) prior to or at the index date, while they had to be aged ≥ 40 years at the index date. People aged < 40 years were excluded from the analyses, as both osteoarthritis [27] and Parkinson’s disease [28] are age-related disorders frequently occurring in the second half of life. After applying similar inclusion criteria, individuals without osteoarthritis were matched to those with osteoarthritis using propensity scores based on sex, age and index year. In patients without osteoarthritis, index date corresponded to a randomly selected visit date between January 2000 and December 2016. Finally, the selection of study patients is displayed in Fig. 1.

### 2.3 Study variables

The independent and dependent variables were osteoarthritis and Parkinson’s disease, respectively. Osteoarthritis included polyosteoarthritis (ICD-10: M15), hip osteoarthritis (ICD-10: M16), knee osteoarthritis (ICD-10: M17), first carpometacarpal joint osteoarthritis (ICD-10: M18), and other and unspecified osteoarthritis (ICD-10: M19). Demographic variables included sex and age. Based on previous literature [11], several chronic conditions documented prior to or on the index date were included, and these disorders were hypertension (ICD-10: I10), diabetes mellitus (ICD-10: E10-E14), thyroid diseases (ICD-10: E00-E07), dyslipidemia (ICD-10: E78), coronary heart disease (ICD-10: I24 and I25), gout (ICD-10: M10), chronic obstructive pulmonary disease (ICD-10: J44), stroke including transient ischemic attack (ICD-10: I63, I64 and G45), and intracranial injury (ICD-10: S06).

### 2.4 Incidence of Parkinson’s disease

This study analyzed the 10-year cumulative incidence of Parkinson’s disease in patients with and without osteoarthritis. Participants were followed up until the date on which Parkinson’s disease was diagnosed or the last visit date within the 10 years following the index date.

### 2.5 Statistical analyses

Baseline characteristics (i.e., sex, age and chronic conditions) were compared between patients with and without osteoarthritis using McNemar tests for all variables except the continuous age variable (Wilcoxon signed-rank test). In addition, the 10-year incidence of Parkinson’s disease in the groups with and without osteoarthritis was studied using Kaplan-Meier curves and the log-rank test. Finally, unadjusted and adjusted Cox regression analyses were conducted to investigate the association between osteoarthritis and the incidence of Parkinson’s disease. The osteoarthritis-Parkinson’s disease relationship was studied in the overall population and in sex (i.e., female and male) and age subgroups (i.e., ≤50, 51–60, 61–70, 71–80, and > 80 years), while this association was also stratified by the type of osteoarthritis (i.e., polyosteoarthritis, hip osteoarthritis, knee osteoarthritis, and other and unspecified osteoarthritis). As only a small proportion of patients had first carpometacarpal joint osteoarthritis, the association between this type of osteoarthritis and Parkinson’s disease could not be investigated. Adjusted models simultaneously included all chronic conditions mentioned above. Given that sex and age were used to match patients without osteoarthritis to those with osteoarthritis, these two demographic variables were not included in the regression models. Results from the Cox regression analyses are presented as HRs with 95% confidence intervals (CIs). P-values < 0.050 were considered statistically significant. All analyses were performed using SAS 9.4.

## 3 Results

**Fig. 2:**
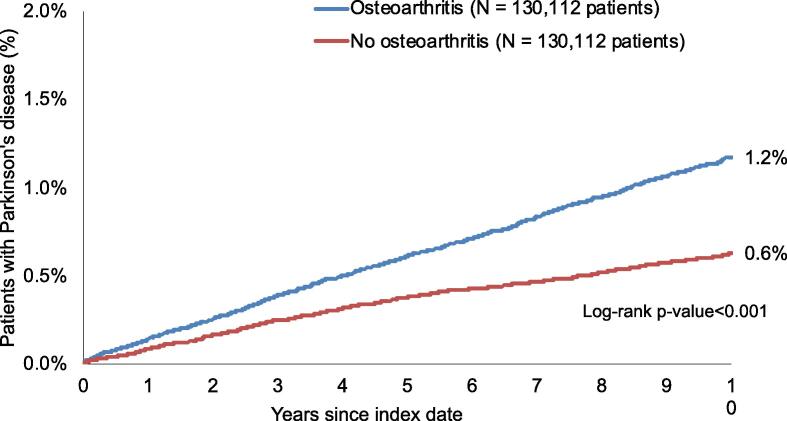
Cumulative incidence of Parkinson’s disease in patients with and without osteoarthritis followed up in general practices in the United Kingdom for up to 10 years.

There were 260,224 patients included in this study (62.0% women; mean [standard deviation] age 66.4 [12.7] years) (Table 1). The three most frequent disorders in the sample were hypertension (32.4% in the osteoarthritis group and 28.3% in the no osteoarthritis group), diabetes mellitus (9.3% and 8.2%, respectively) and thyroid diseases (9.1% and 7.5%, respectively). The different types of osteoarthritis were polyosteoarthritis (6.3%), hip osteoarthritis (5.9%), knee osteoarthritis (5.1%), first carpometacarpal joint osteoarthritis (0.1%), and other and unspecified osteoarthritis (82.6%). Kaplan-Meier curves are displayed in Fig. 2. Within 10 years of the index date, 1.2% of patients with osteoarthritis and 0.6% of their counterparts without osteoarthritis were diagnosed with Parkinson’s disease (log-rank p-value < 0.001). Table 2 shows the results of the unadjusted and adjusted Cox regression analyses. After adjusting for chronic conditions, osteoarthritis was positively and significantly associated with the 10-year incidence of Parkinson’s disease in the overall population (HR = 1.82, 95% CI: 1.63–2.02). Similar findings were obtained in sex and age subgroups, with HRs ranging from 1.59 (95% CI: 1.27–1.99) in people aged > 80 years to 3.22 (95% CI: 1.06–9.38) in those aged ≤ 50 years. In terms of the different types of osteoarthritis, only knee (HR = 2.32, 95% CI: 1.42–3.79) and other and unspecified osteoarthritis (HR = 1.85, 95% CI: 1.64–2.08) were significantly associated with the odds of being diagnosed with Parkinson’s disease (Table 3).

**Table 1:** Baseline characteristics of study patients after 1:1 matching.

Variable	Patients with osteoarthritis (N = 130,112)	Patients without osteoarthritis (N = 130,112)	P-value
*Sex*			
Female	80,687 (62.0)	80,687 (62.0)	1.000
Male	49,425 (38.0)	49,425 (38.0)	

*Age (in years)*			
Mean (standard deviation)	66.4 (12.7)	66.4 (12.7)	1.000
≤50	14,544 (11.2)	14,544 (11.2)	1.000
51–60	31,170 (24.0)	31,170 (24.0)	
61–70	34,217 (26.3)	34,217 (26.3)	
71–80	30,047 (23.1)	30,047 (23.1)	
>80	20,134 (15.4)	20,134 (15.4)	

*Disorders documented prior to or on the index date*			
Hypertension	42,170 (32.4)	36,776 (28.3)	<0.001
Diabetes mellitus	12,114 (9.3)	10,660 (8.2)	<0.001
Thyroid diseases	11,781 (9.1)	9,760 (7.5)	<0.001
Dyslipidemia	11,031 (8.5)	9,240 (7.1)	<0.001
Coronary heart disease	8,972 (6.9)	6,347 (4.9)	<0.001
Gout	5,952 (4.6)	4,601 (3.5)	<0.001
Chronic obstructive pulmonary disease	5,865 (4.5)	5,339 (4.1)	<0.001
Stroke including transient ischemic attack	5,759 (4.4)	5,964 (4.6)	0.053
Intracranial injury	1,811 (1.4)	2,015 (1.5)	0.009

Data are N (%) unless otherwise specified.

**Table 2:** Association between osteoarthritis and the incidence of Parkinson’s disease in the overall population and in sex and age subgroups.

Population	Incidence of Parkinson’s disease in patients with osteoarthritis^a^	Incidence of Parkinson’s disease in patients without osteoarthritis^a^	Unadjusted hazard ratio (95% confidence interval)	P-value	Adjusted hazard ratio (95% confidence interval)^b^	P-value
Overall	124.1	67.1	1.85 (1.66–2.06)	<0.001	1.82 (1.63–2.02)	<0.001

*By sex*						
Female	101.1	52.6	1.92 (1.65–2.24)	<0.001	1.88 (1.61–2.19)	<0.001
Male	161.8	90.8	1.78 (1.53–2.07)	<0.001	1.75 (1.51–2.04)	<0.001

*By age (in years)*						
Age ≤ 50	14.7	4.2	3.48 (1.15–10.58)	0.028	3.22 (1.06–9.38)	0.040
Age 51–60	39.1	17.9	2.18 (1.47–3.24)	<0.001	2.12 (1.43–3.14)	<0.001
Age 61–70	116.3	54.1	2.15 (1.72–2.68)	<0.001	2.14 (1.72–2.67)	<0.001
Age 71–80	243.7	136.8	1.78 (1.52–2.09)	<0.001	1.79 (1.53–2.09)	<0.001
Age > 80	218.3	139.8	1.56 (1.24–1.96)	<0.001	1.59 (1.27–1.99)	<0.001

a Number of patients diagnosed with Parkinson’s disease per 100,000 people.

b Adjusted Cox regression models included hypertension, diabetes mellitus, thyroid diseases, dyslipidemia, coronary heart disease, gout, chronic obstructive pulmonary disease, stroke including transient ischemic attack, and intracranial injury.

**Table 3:** Association between different types of osteoarthritis and the incidence of Parkinson’s disease in the overall population.

Type of osteoarthritis	Incidence of Parkinson’s disease in patients with osteoarthritis^a^	Incidence of Parkinson’s disease in patients without osteoarthritis^a^	Unadjusted hazard ratio (95% confidence interval)	P-value	Adjusted hazard ratio (95% confidence interval)^b^	P-value
Polyosteoarthritis	105.3	65.5	1.61 (1.03–2.51)	0.038	1.52 (0.97–2.39)	0.066
Hip osteoarthritis	123.7	85.8	1.44 (0.95–2.18)	0.083	1.41 (0.93–2.13)	0.104
Knee osteoarthritis	163.7	71.3	2.30 (1.40–3.75)	<0.001	2.32 (1.42–3.79)	<0.001
Other and unspecified osteoarthritis	123.6	65.7	1.88 (1.67–2.11)	<0.001	1.85 (1.64–2.08)	<0.001

The reference group is the group without osteoarthritis.

a Number of patients diagnosed with Parkinson’s disease per 100,000 people.

b Adjusted Cox regression models included hypertension, diabetes mellitus, thyroid diseases, dyslipidemia, coronary heart disease, gout, chronic obstructive pulmonary disease, stroke including transient ischemic attack, and intracranial injury.

## 4 Discussion

### 4.1 Main findings

In this retrospective cohort study of more than 260,200 patients followed in around 260 general practices from the United Kingdom, the 10-year cumulative incidence of Parkinson’s disease was 1.2% and 0.6% in those with and without osteoarthritis, respectively. These findings were corroborated in the adjusted Cox regression analyses, as osteoarthritis was positively and significantly associated with the incidence of Parkinson’s disease in the overall population and all sex and age subgroups. Sensitivity analyses also revealed that, in terms of the type of osteoarthritis, there was a significant relationship of knee osteoarthritis and other and unspecified osteoarthritis with Parkinson’s disease. To the best of the authors’ knowledge, this is one of the first studies on the potential association between osteoarthritis and Parkinson’s disease, while it is the study with the largest sample size to date.

### 4.2 Interpretation of the findings

This UK study revealed that osteoarthritis was associated with a significant increase in the incidence of Parkinson’s disease within the 10 years following the initial diagnosis of osteoarthritis. This finding is in line with previous research reporting a positive osteoarthritis-Parkinson’s disease relationship in Taiwan, although the HR of the association was slightly higher in this study than in the previous study (1.82 versus 1.41) [11]. The difference in the strength of the association between osteoarthritis and Parkinson’s disease between these two studies may be explained by substantial differences in the epidemiology of osteoarthritis between the United Kingdom and Taiwan. Indeed, in a systematic analysis of the Global Burden of Disease Study 2017, age-standardized years lived with disability rates of osteoarthritis were approximatively twice as high in the United Kingdom than in Taiwan [25]. In this context, the deleterious effects of osteoarthritis on Parkinson’s disease may be stronger in the United Kingdom than in Taiwan. Another interesting finding of this study was that knee osteoarthritis and other and unspecified osteoarthritis, but not polyosteoarthritis and hip osteoarthritis, were associated with a significant increase in the risk of Parkinson’s disease. Given that all HRs were positive and that 95% CIs were relatively large, it is possible that these sensitivity analyses lacked statistical power, and more data are therefore warranted to better characterize these potential differential associations.

The association between osteoarthritis and Parkinson’s disease may involve common risk factors as well as several mediating factors. One risk factor likely playing a significant role in the etiopathogenesis of both osteoarthritis and Parkinson’s disease is inflammation. Inflammatory pathways in osteoarthritis are multiple, and these pathways include the modulation of pain responses, the development of a local and low-grade inflammation of the synovial tissue, and the regulation of the catabolic response of chondrocytes [14]. In Parkinson’s disease, inflammation is characterized by several cellular and molecular phenomena such as release of pro-inflammatory cytokines by microglial cells, increased expression of adhesion molecules by the endothelium and recruitment of T cells into the brain [15]. Another risk factor for osteoarthritis and Parkinson’s disease is vitamin D deficiency, a deficiency mainly caused by limited sun exposure and highly prevalent in the world [29]. Although the effects of vitamin D deficiency on osteoarthritis remain insufficiently understood, vitamin D is known to promote chondrocyte hypertrophy via its receptors, and lower levels of vitamin D may thus be associated with an alteration of the homeostasis of the articular cartilage and a decrease in cartilage thickness [17]. Interestingly, vitamin D also has neuroprotective properties, and previous research has found a positive association between low levels of vitamin D, Parkinson’s disease and the severity of the motor symptoms of this neurodegenerative disorder [18].

Beside these two common risk factors, the association between osteoarthritis and Parkinson’s disease may involve several mediating factors such as physical inactivity (i.e., not meeting physical activity recommendations), hypertension and depression. As physical activity may transitorily increase pain and disability, and as fear that physical activity may aggravate osteoarthritis-related lesions is fairly common, physical activity recommendations are less frequently met in people with osteoarthritis than in the general population [30]. Meanwhile, there is strong evidence pointing towards the existence of an inverse dose–response association between physical activity and Parkinson’s disease, potentially explained by the fact that physical activity leads to an increase in the production of several growth factors and a decrease in oxidative stress [20]. Interestingly, some recent data tend to suggest that osteoarthritis is also a risk factor for hypertension [21], while hypertension may favor the occurrence of Parkinson’s disease via hypertensive vasculopathy of basal ganglia [22]. Since the present Cox regression analyses were adjusted for hypertension but not for its potential complications, residual confounding is possible and hypertension may still be involved in the associations observed in this study. Finally, individuals diagnosed with osteoarthritis are at a significantly higher risk for depression than their counterparts without osteoarthritis, and the effects of osteoarthritis on mental health may be mediated by pain and disability [23]. In turn, there is a significant association between depression and the incidence of Parkinson’s disease, with these two disorders sharing common physiopathological characteristics (e.g., cerebral atrophy and decreased levels of gamma-aminobutyric acid) [24].

### 4.3 Public health implications and directions for future research

Based on the results of this retrospective cohort study, osteoarthritis is associated with an increase in the incidence of Parkinson’s disease. In this context, measures aiming at the prevention of Parkinson’s disease should be implemented in patients with osteoarthritis. One of these measures could be physical activity (e.g., walking, running and yoga), as an important body of literature has shown that physical activity can decrease the risk of Parkinson’s disease and alleviate the symptoms of this neurodegenerative condition [31]. In addition, the management of depression and impaired mental wellbeing in people with osteoarthritis may be of utmost importance, and this management may consist of patient education programs, cognitive behavioral therapy and pharmacological interventions [32]. Finally, more studies of longitudinal nature are needed to corroborate or invalidate these findings in other settings and countries, while further research should be conducted to better characterize the mechanisms underlying the osteoarthritis-Parkinson’s disease relationship.

### 4.4 Strengths and limitations

Two major strengths of this study are the large sample size and the use of longitudinal data. Nonetheless, this study also displays several limitations that should be acknowledged at this point. First, the diagnosis of osteoarthritis relied on ICD-10 codes only, and more information on clinical and radiographic characteristics of the disorder may have allowed for more detailed analyses. Second, patients may have been diagnosed with Parkinson’s disease in neurological practices, and the incidence of this neurodegenerative condition may have been underestimated. Third, no data was available on health behaviors (e.g., smoking and physical activity), and as some of these behaviors are associated with both osteoarthritis and Parkinson’s disease [19], [20], [33], [34], this may have introduced some bias in the analysis.

## 5 Conclusions

The present retrospective cohort study of 260,224 patients followed in general practices in the United Kingdom for up to 10 years showed that the cumulative incidence of Parkinson’s disease was significantly higher in those with than in those without osteoarthritis (1.2% versus 0.6%). These results were corroborated in the adjusted Cox regression analyses, where osteoarthritis was associated with a 1.82-fold increase in the risk of Parkinson’s disease. Further longitudinal studies are needed to investigate the effects of osteoarthritis on Parkinson’s disease in other settings, as well as factors playing a potential mediating role in this relationship.

### CRediT authorship contribution statement

**Louis Jacob:** Conceptualization, Methodology, Writing – original draft. **Lee Smith:** Writing – review & editing. **Ai Koyanagi:** Writing – review & editing. **Alexis Schnitzler:** Writing – review & editing. **Jae Il Shin:** Writing – review & editing. **Karel Kostev:** Conceptualization, Methodology, Supervision, Writing – review & editing, Investigation.

## Declaration of Competing Interest

The authors declare that they have no known competing financial interests or personal relationships that could have appeared to influence the work reported in this paper.
